# Lack of Public Awareness and Prehospital Care in Severe Trauma Patients: The Weakest Link in the Indian Healthcare System

**DOI:** 10.7759/cureus.61208

**Published:** 2024-05-28

**Authors:** Akash Singhal, Gourav Sharma, Dina Shah, Shruti Dubey, Madhur Kalra, Shehtaj Khan

**Affiliations:** 1 Critical Care Medicine, Max Sahara Hospital, Lucknow, IND; 2 Orthopaedics, Chirayu Medical College and Hospital, Bhopal, IND; 3 Emergency Medicine, Visionnaire Emergency Care Services, Noida, IND; 4 Emergency Medicine, LN Medical College and Research Center, Bhopal, IND; 5 Emergency Medicine, Alchemist Hospital, Panchkula, IND

**Keywords:** acls, bls, healthcare system, prehospital care, awareness, ems, emergency medical services, trauma

## Abstract

Introduction: Management of trauma involves both in-hospital and prehospital care. The level of prehospital care plays a vital role in trauma management. Low- and middle-income countries are still in the nascent stages of development of their emergency medical services (EMS) systems. Also, there have been insufficient studies assessing the availability and level of prehospital care in developing nations such as India. Therefore, we decided to study the level of awareness and prehospital care given to severe trauma patients.

Materials and methods: We conducted this prospective observational study at the emergency department of Fortis Hospital, Noida, Uttar Pradesh, in Northern India. All adults between ages 18 and 85 years presenting with severe trauma (immediate life- or limb-threatening conditions requiring emergent intervention) were included. We measured the primary outcome in terms of why people did not avail EMS. We measured secondary outcomes in terms of intervention done in patients coming to us via EMS.

Results: Out of 101 patients, 89 (88.12%) were transported to Fortis Hospital through non-EMS, whereas only 12 (11.88%) patients were transported by EMS. We found the difference to be statistically significant. The major reason given for not summoning advanced trauma care services in patients was a lack of awareness about the potential benefits of EMS (n = 64 [72%]), followed by a lack of availability (n = 24 [27%]), and financial reasons (n = 1 [1.1%]).

Conclusion: We conclude that the level of awareness about EMS for severe trauma patients was found to be low in our study. There is a need for an awareness-creation program across the nation to fill this gap.

## Introduction

Injury is the fourth leading cause of global death [1]. A further 40% increase in trauma deaths is expected by 2030 [1]. Almost 90% of injury deaths occur in low-and middle-income countries (LMIC) [1]. If death rates of severe trauma patients could be reduced to match the level of high-income countries, about 1,730,000 to 1,965,000 lives could be saved in LMIC [2]. However, there has been little research on injuries emerging from LMIC [3].

Emergency medical services (EMS) can be defined as “a comprehensive system which provides the arrangements of personnel, facilities and equipment for the effective, coordinated and timely delivery of health and safety services to victims of sudden illness or injury” [4,5]. The delivery of EMS in prehospital settings can be categorized broadly into Franco-German or Anglo-American models [4,5].

Another way to classify EMS is according to the level of service and scope of practice provided. These are usually classified as basic life support (BLS) or advanced life support (ALS) levels. BLS is tightly associated with the “load and go” philosophy, providing noninvasive basic interventions and rapid transport to a definitive healthcare facility. ALS is a more advanced approach that along with the basic interventions has both invasive and pharmaceutical capabilities [4,6].

Emergency medical services in India

EMS has changed since it was commonly stated that “EMS systems in India are best described as fragmented” [7,8]. The establishment of EMS in India was not a nationwide movement; rather, it started in Mumbai city in 1985 when 15 ambulances were connected to a central wireless dispatch center by the Association for Trauma Care of India. This typically exemplifies the development of EMS in India, which has not had a transformative moment like the United States. It was driven by passionate individuals and organizations dedicated to changing the “transport vehicle concept of ambulances” to “lifesaving emergency medical transportation” [7].

India has adopted primarily the older “scoop and run” model. The provision of emergency services is enshrined in India’s constitution. If any hospital does not provide timely medical treatment to a person, it is considered a violation of the person’s “right to life” as guaranteed under Article 21 of India’s constitution [7,8]. Although India has the fastest-growing population and an ambitious growth aspiration, it has always had a disproportionately small health budget [7,9]. In 2015, India's expenditure on health was 1.2% of gross domestic product (GDP) compared to 3% and 8.3% of China and the United States, respectively [9]. Almost 23% of all trauma that occurs in India is related to transportation, with 1,374 accidents and 400 deaths taking place every day on Indian roads. The rest of the 77.2% of trauma is related to other events such as falls, drowning, agriculture-related injuries, and burns [7,10].

In our emergency department at Fortis Hospital, we see 2,000 to 4,000 trauma patients in one year, of which 10%-15% are severe. As a tertiary care center in western Uttar Pradesh, we receive patients transported from accident scenes and primary as well as secondary care centers in the surrounding regions. Only a few of these patients receive primary care before arriving at our facility. Foreign literature has often focused on how patients are managed in prehospital settings and the impact of this management on patient mortality and morbidity. Researchers have collected much data on injuries and their implications, but there have been no studies assessing the availability and level of prehospital care in developing nations such as India.

Furthermore, Indian studies are lacking in the field of emergency medicine. Although researchers have come far, the available studies are poor in quality and inadequate for properly informing health policy [7,11]. We are still in the nascent stages of the development of emergency medicine; therefore, we decided to study the level of public awareness and prehospital care given to severe trauma patients in Northern India.

Primary objective

Our primary objective was to assess the causes for not availing advanced trauma care ambulance services in severe trauma patients; the reasons could be the nonavailability of EMS and lack of awareness about the potential benefits of EMS as well as the financial burden on the patient’s family. Our secondary objective was to assess the level of prehospital care given en route by BLS and advanced life support (ALS) ambulance services before arriving at a definitive care center.

## Materials and methods

This is a prospective observational study conducted in the emergency department of Fortis Hospital, Noida, Uttar Pradesh in Northern India. We conducted this research from April 2016 to March 2017 after obtaining approval from the Institutional Ethics Committee (IEC).

Inclusion and exclusion criteria

Inclusion criteria included severe trauma patients (both men and women) between ages 18 and 85. Exclusion criteria included burn patients or patients who had received treatment in the emergency department of another hospital for more than four hours.

Sample size

The sample size was calculated using the formula:

$n = \frac{Z^2pq}{d^2}$

where n = sample size, Z = value at two-sided confidence interval, p = prevalence in %, q = 100 - p, and d = margin of error.

The prevalence of awareness of the use of EMS was 50% (through a pilot study on 16 cases). The two-sided confidence interval was 95% with 20% relative error in the given prevalence, and the sample size was 97. Hence, our goal was to include at least 100 patients.

Study method

We included all adults between 18 and 85 years presenting with severe trauma (immediate life- or limb-threatening conditions requiring emergent intervention). Written informed consent in the local language was taken from each patient. We recorded demographics in terms of age and sex of all patients. We applied the exclusion criteria and selected study cases and further subdivided them into those coming through EMS and those coming through non-EMS.

Primary outcome

We measured the primary outcome by investigating why people did not avail EMS in cases of severe trauma. This was evaluated by verbally asking the patient and patient’s attendant about their reasons for not availing EMS in terms of nonavailability of EMS, lack of awareness among people about the potential benefits of EMS, or financial issues preventing the patient or patient’s family from availing EMS.

Secondary outcome

We measured the secondary outcome by investigating the interventions performed on patients arriving to us via EMS. Interventions performed by BLS services were noted for measurement of on-route vitals, cervical spine immobilization, fluid resuscitation en route, and oxygen (O_2_) therapy. Interventions performed by ALS services were noted for all parameters of BLS services, including a primary survey using the Airway Breathing Circulation Disability (ABCD) approach and any intervention as required in terms of endotracheal intubation, spinal immobilization, drug administration, and blood given.

Statistical method

Categorical variables were expressed in frequency and percentages, and continuous variables were expressed in mean with standard deviations. We analyzed associations between two variables to test the difference in proportions using chi-square tests; otherwise, we used Fisher’s exact test. We used the Kruskal-Wallis H test to compare the median scores among the three groups. We used SPSS version 22 (IBM Corp., Armonk, NY) for data analysis. We have considered a p-value < 0.05 as statistically significant.

## Results

**Figure 1:**
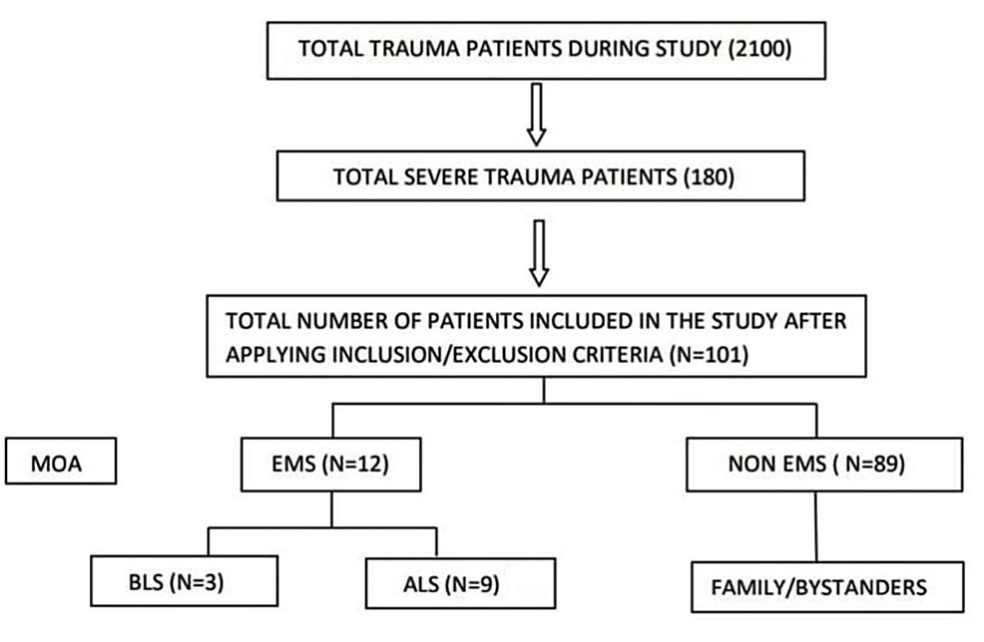
Distribution of trauma patients and mode of arrival MOA: Mode of arrival; EMS: Emergency medical services; BLS: Basic life support; ALS: Advanced life support.

In our study period of April 1, 2016, to March 30, 2017, a total of 2,100 patients presented to our emergency department with trauma, out of which 180 patients had severe trauma. After applying inclusion criteria and exclusion criteria, we included 101 patients in this study (Figure 1).

Among 79 excluded patients, 11 (13.92%) patients were <18 years old; 66 (83.54%) stayed in another hospital for >4 hours, and two (2.53%) patients arrived with severe burns.

The average age of patients was 39.02 years (SD = 20.8) in our study. There was a significant association between gender distribution and severe trauma patients with p-value < 0.001, the majority of whom were men (n = 86 [85.10%]). In our study, 89 (88.11%) patients were brought through non-EMS, whereas only 12 (11.88%) patients came through EMS, which was found to be statistically significant with a p-value < 0.001 as shown in Table 1.

**Table 1:** Demography and MOA of the patients This table shows the age, sex, and MOA of the patients (n = 101). MOA: Mode of arrival; EMS: Emergency medical services; BLS: Basic life support; ALS: Advanced life support.

Parameters	Value	p-value
Age (years)	39.02 ± 17.28 (Mean ± SD)	-
Age range (years)	18-85 (Min-Max)	-
Male–female ratio	86:15	<0.001
Mode of arrival (EMS-Non-EMS)	12:89	<0.001

Those coming through EMS were further categorized into patients who came via BLS services (n = 3) and patients who came via ALS services (n = 9). Mechanism of injury was predominantly road traffic accidents in 73 (72.2%) patients, followed by fall from height in 19 (18.8%) patients, and assault in only nine (8.9%) patients.

We used the Kruskal-Wallis H test to compare the median arrival time, and we found that time to door was significantly longer in patients who came through both BLS and ALS services in comparison to patients coming through non-EMS (Table 2).

**Table 2:** Time to door (in minutes) This table shows time to door (in minutes), which is the time lapse between the time of the incident and the time of arrival. EMS: Emergency medical services; BLS: Basic life support; ALS: Advanced life support.

Mode	Median (interquartile range)	Min-Max	*p-value
ALS	180 (130-208)	75-240	<0.001 (Kruskal-Wallis H test*)
BLS	95 (70-210)	70-210	
Non-EMS	45 (30-88)	5-480	

Primary outcome

**Figure 2:**
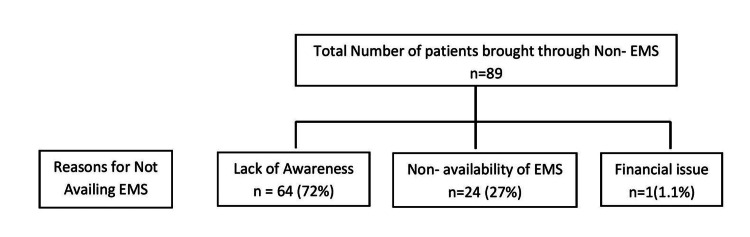
Primary outcome for not availing EMS EMS: Emergency medical services.

Lack of awareness was the main reason for not availing prehospital care (n = 64[72%]), followed by nonavailability of EMS (n = 24 [27%]) as the second most common cause, and financial issues in one patient (1.1%) (Figure 2).

Secondary outcome

Patients coming through either ALS or BLS services received prehospital care when compared to those coming through non-EMS, and the difference was statistically significant (p < 0.05).

Out of 89 patients coming with non-EMS, 26 (29.2%) were in shock. One out of three patients availing BLS services was found to be in shock when compared to patients who received ALS care, although it was statistically insignificant (p > 0.05). This was primarily because we had relatively fewer (smaller sample size) patients coming through ALS services when compared to non-EMS. All patients availing BLS services had their vitals checked en route and were given intravenous fluids; two (66.7%) of them received c-spine immobilization, and two (66.7%) received oxygen therapy (Table 3).

**Table 3:** Intervention done in patients arriving via BLS services C-spine: Cervical spine; BLS: Basic life support.

BLS care given (n = 3)	Yes (%)
Prehospital vital monitoring	3 (100.0)
Fluid administration	3 (100.0)
C-spine immobilization	2 (66.7)
O_2 _therapy	2 (66.7)

All patients coming through ALS received BLS care and were also assessed in terms of the ABCD approach and stabilized accordingly (Tables 4, 5).

**Table 4:** Primary survey (ABCD) This table shows a primary survey done on patients arriving via ALS services. ABCD: Airway Breathing Circulation Disability; ALS: Advanced life support.

ALS given (n = 9)	Yes (%)
Airway	9 (100.0)
Breathing	9 (100.0)
Circulation	9 (100.0)
		Disability	9 (100.0)

**Table 5:** ALS interventions This table shows various interventions done in patients coming via ALS services. ALS: Advanced life support.

ALS interventions	N (%)
Endotracheal intubation	7 (77.8)
Spinal immobilization	9 (100)
Drug administration	9 (100)
Blood administration	1 (11.1)

## Discussion

In this prospective observational study, 101 patients with severe trauma were enrolled. We further divided patients into those who availed EMS and those who came through non-EMS.

The mean age was 39 years. There were almost three times more men as compared to women; this was in concordance with the study by Paravar et al., whereas a study by Carr et al. showed an almost equal sex ratio [1,12]. This might be attributed to the fact that the Carr et al. study was done in the United States, which has a more equalized ratio of female to male drivers when compared to both Iran and India; both are developing countries with fewer female drivers as well as male drivers being more aggressive and impulsive, thereby being more prone to injuries [12].

Emergency medical services versus nonemergency medical services

When compared to a study by Cornwell et al. in the United States in which 65% of people came through EMS, the majority of people (n = 89 [88.1%]) in our study came through non-EMS [13]. This might be because ambulance services commonly cater to a locality or a community in the United States. They have self-dispatching ambulances available in the community and a national emergency number (i.e., 911) that works for all three emergency services (i.e., police, ambulance, and fire). In most cases, a 911 call will be answered at a central facility, usually referred to as a Public Safety Answering Point (PSAP), after which the needs of the caller are identified, and the call is routed to the dispatcher for the emergency service(s) required [14].

In India, EMS still remains largely fragmented and confined to larger cities and patients who can pay out of pocket in the majority of cases. India has two helpline numbers: 108 and 102, both being publicly funded ambulance systems; together, they run more than 17,000 ambulances across India. In 2005, 108 was introduced as a toll-free telephone number for emergency response services. It is a national emergency number for emergency medical services [15]. Also, private hospitals have their own telephone numbers for ambulance services, unlike developed countries [7].

The median time to door in our study was 180 minutes in the case of patients arriving through ALS, 95 minutes through BLS, and 45 minutes through non-EMS, whereas in the study by Carr et al., median duration in minutes for urban, suburban, and rural ground ambulances for the total prehospital interval was 30.96, 30.97, and 43.17 minutes, respectively [12]. The mean transport and time to door were 11.1 and 12.8 minutes, respectively. This reflects the need to improve the transportation in India's system.

In our study, the major reason for people not availing EMS was lack of awareness in 64 (72%) patients, followed by nonavailability of ambulance in 24 (27%) patients, and financial issues in one patient. In a survey conducted in Maharashtra, researchers found that 291 (23.8%) people were not aware of any EMS available. Of the 929 (76.2%) who are aware of dialing 108 in case of a medical emergency, two-thirds were unaware of the development of the unified emergency number (112) [16]. The reason for better awareness in Modi et al.'s study compared to the current study is that most responders were from the city of Mumbai. Nonetheless, the lack of awareness about EMS is a major issue that needs to be addressed. These numbers might be worse in rural areas, where the presence of EMS is almost negligible, making their availability an even bigger issue.

Along with the development of EMS, quality and standardization in the management of acute trauma cases are also important. The advanced trauma life support (ATLS) program is designed by the American College of Surgeons for medical providers, which provides a systematic approach to the initial assessment and management of acute trauma cases [17]. Such courses should be introduced to the training of all emergency department physicians and medical providers to improve trauma care in India.

Limitations

Although we included all severe trauma patients arriving to us within four hours through EMS to avoid those who had already received primary care at other hospitals in in-hospital settings, we still included patients arriving to us who were seen at some primary care centers and then transported to our hospital within the first four hours. We were unable to obtain adequate numbers of patients coming via EMS services. This can be attributed to both the lack of availing EMS services among the general population and the fact that our hospital is a private institution, where the number of patients is less compared to a government institution.

## Conclusions

The major reason for not availing the advanced trauma care services was a lack of awareness about the potential benefits of EMS in 64 (72%) patients. This was followed by the nonavailability of EMS (n = 24 [27%]) and financial reasons (n = 1 (1.1%]). Therefore, our study concludes that the level of awareness about EMS among severe trauma patients is low. There is a need for an awareness-creation program across India to fill this gap. Further, there is a need for a centralized medical authority, which would be responsible for preparing protocols and imparting technical assistance, training, capacity building, and accreditation of emergency service procedures.
